# An analysis of the relationship of selected personality traits on perceived stress in patients undergoing ablation for atrial fibrillation and atrial flutter

**DOI:** 10.3389/fpsyt.2026.1681576

**Published:** 2026-08-10

**Authors:** Adrianna Falenta, Natalia Przytocka, Natalia Droździok, Szymon Florek, Alexander Suchodolski, Magdalena Piegza, Mariola Szulik, Zbigniew Kalarus, Piotr Gorczyca, Paweł Dębski

**Affiliations:** 1 Student’s Research Group, Department of Psychiatry, Faculty of Medical Sciences in Zabrze, Medical University of Silesia in Katowice, Katowice, Poland; 2 Department of Psychoprophylaxis, Faculty of Medical Sciences in Zabrze, Medical University of Silesia in Katowice, Katowice, Poland; 3 Doctoral School Medical University of Silesia, Faculty of Medical Sciences (FMS) in Zabrze Medical University of Silesia in Katowice, Katowice, Poland; 4 Department of Cardiology and Electrotherapy, Silesian Center for Heart Diseases, Faculty of Medical Sciences (FMS) in Zabrze Medical University of Silesia in Katowice, Katowice, Poland; 5 Department of Psychiatry, Faculty of Medical Sciences in Zabrze, Medical University of Silesia in Katowice, Katowice, Poland; 6 Department of Medical and Health Sciences, WSB University Faculty of Applied Sciences, Dąbrowa, Górnicza, Poland

**Keywords:** ablation, cardiology, dark tetrad, psychiatry, stress

## Abstract

**Introduction:**

The aim of this cross-sectional study was to assess the correlation between selected personality traits and the level of perceived stress in patients undergoing catheter ablation for atrial fibrillation or atrial flutter. These arrhythmias are among the most common cardiac rhythm disorders. Previous research suggests that psychological factors such as stress and personality traits may correlate their development and clinical course. Understanding these relationships may improve the effectiveness of patient care, particularly in terms of psychological support during the perioperative period.

**Methods:**

The study was conducted over a five-month period in a cardiology department. A total of 50 patients were included in the study sample. Psychological characteristics and stress levels were assessed at a single time-point using a custom-designed questionnaire and three standardized psychometric tools: PSS-10, SD4 and DS14.

**Results:**

Statistical analysis revealed a positive correlation between perceived stress and elevated levels of psychopathy, social inhibition and negative affectivity. Gender-stratified analyses showed that men scored higher on sadism, while women reported higher levels of stress, negative affectivity and social inhibition. Participants who reported greater concern regarding body weight management exhibited increased Machiavellianism levels, whereas other physical health behaviours were more frequently neglected among individuals with stronger Type D personality traits.

**Conclusions:**

The study indicates a potential correlation between dark personality traits and elevated stress levels prior to ablation procedures. Nonetheless, the evidence remains inconclusive. Future research should incorporate exclusion criteria, direct body weight measurement and the inclusion of control group.

## Graphical Abstract

**Figure:**
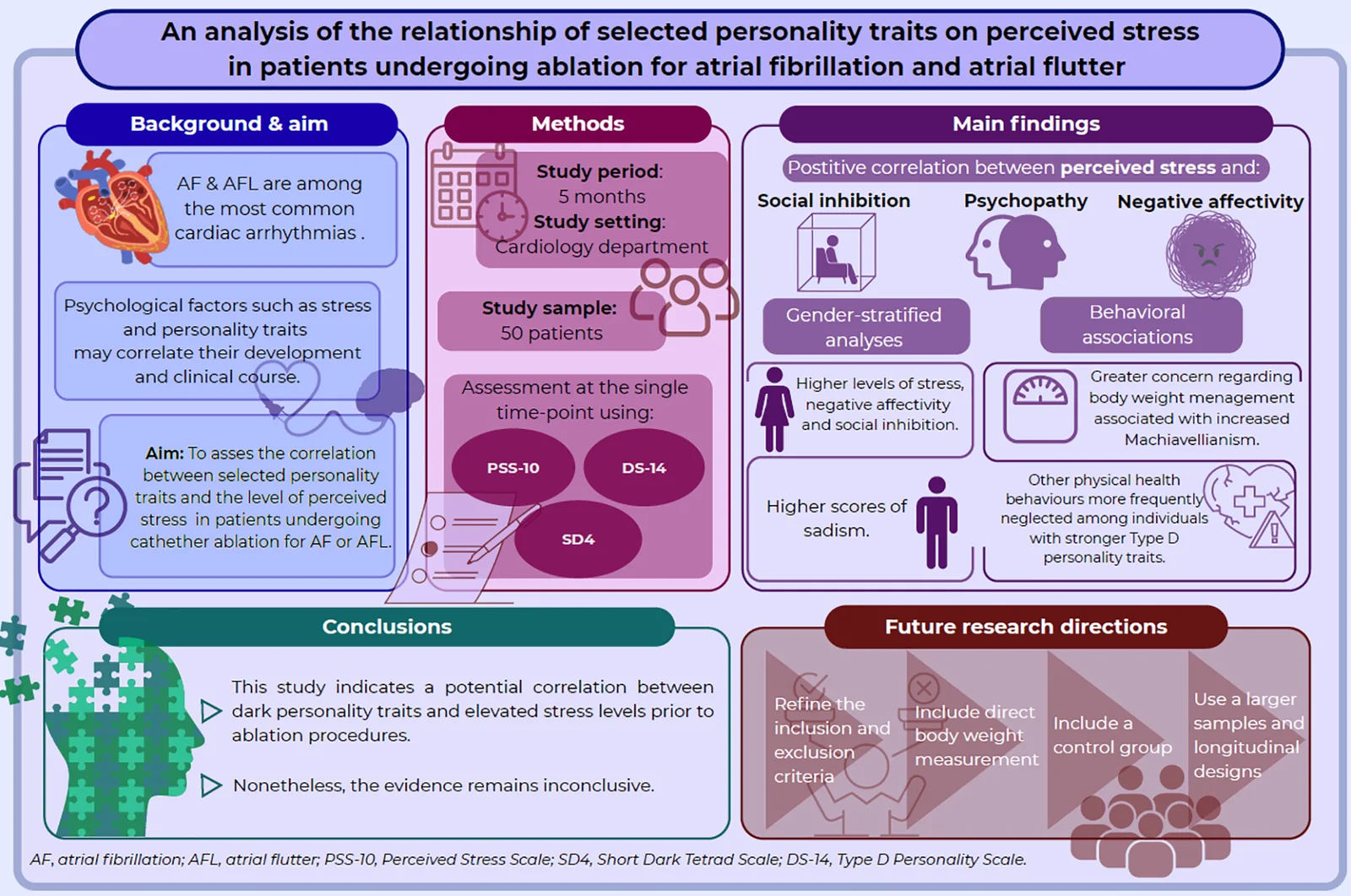

## Introduction

Psychocardiology is an interdisciplinary field of science and clinical practice that focuses on analyzing the mutual relationships between psychological factors and the functioning of the cardiovascular system. It is widely accepted that factors such as stress, anxiety, depression, and specific personality traits may be associated with an increased risk of developing heart disease as well as with its unfavorable course. The pathophysiological mechanisms underlying these associations can be divided into two categories: behavioral mechanisms, which include adverse health behaviors (e.g., unhealthy diet and smoking), and direct physiological mechanisms, such as activation of the hypothalamic-pituitary-adrenal (HPA) axis or the sympathetic nervous system in response to stress (1).

Psychological factors and personality traits are increasingly recognized as important contributors to cardiovascular disease outcomes and patients’ emotional functioning (2).

In the 1990s, Johan Denollet introduced the concept of Type D personality, which encompasses two core components: negative affectivity (NA) – the tendency to experience intense negative emotions such as anxiety, sadness, irritability, or tension, regardless of external circumstances – and social inhibition (SI), defined as a tendency to suppress emotional expression and avoid interpersonal interactions due to fear of disapproval or rejection (3). Research by Kupper and Denollet demonstrated that Type D personality is associated with higher levels of depressive and anxiety symptoms, impaired psychosocial functioning, reduced quality of life and worse clinical outcomes in patients with cardiovascular disease. However, it is important to note that these associations are not direct but rather mediated by multiple factors, including levels of social support and the presence of comorbid affective disorders (4).

In recent years, the construct of dark personality traits has received increased attention. In 2002, Paulhus and Williams introduced the concept of the Dark Triad, which consists of three subclinical, antisocial traits: narcissism, Machiavellianism and psychopathy (5). In 2013 Buckels, Jones and Paulhus proposed an extension of this model by introducing a fourth component – sadism – which gave rise to the concept of the Dark Tetrad (6). Research suggests that these traits may co-occur, forming “the dark core” of personality, which promotes manipulative, impulsive and empathy-deficient behavior (7). Machiavellianism is manifested in a cynical perception of interpersonal relationships, an instrumental approach to others, a strong focus on achieving personal goals, as well as a propensity for manipulation (8). Narcissism is characterized by a stable pattern of grandiosity, a belief in one’s own uniqueness and superiority, and an excessive need for admiration accompanied by a deficit in empathy (9). Psychopathy is a trait associated with a persistent disregard for social norms and the rights of others. Psychopathic behaviors typically emerge in childhood or adolescence and persist into adulthood, negatively affecting interpersonal, occupational, and social functioning. Individuals exhibiting high levels of this trait often display impulsivity, a lack of remorse, an inability to learn from negative consequences of their actions, and a tendency toward criminal behavior (10). Sadism refers to the experience of pleasure and satisfaction derived from the physical or psychological suffering of others. In the literature, a distinction is made between vicarious sadism, which involves deriving enjoyment from observing or imagining others’ suffering, and direct sadism, which encompasses active behaviors aimed at causing harm (11).

Despite growing interest in the field of psychocardiology, relatively few studies have focused on analyzing the relationship between personality structure and the subjectively experienced level of stress in patients undergoing interventional cardiology procedures. These procedures include atrial ablation, which is used to treat atrial fibrillation. This issue represents the central aim of the current study.

Atrial fibrillation (AF) is one of the most common cardiac arrhythmias. Data from the NOMED-AF study, which included over 3,000 individuals aged 65 and older, showed that the prevalence of atrial fibrillation in this age group reaches 19,2% (12). AF is frequently treated with catheter ablation, including pulmonary vein isolation (PVI) (13).Although ablation is an effective treatment for atrial fibrillation and flutter, the procedure itself may pose a significant emotional burden for patients (14). Stress and anxiety are common experiences among cardiology patients in the pre-procedural period (15). Research conducted in the context of coronary angiography and other interventional cardiology procedures suggests that such interventions may be associated with elevated level of perceived stress and anxiety, particularly in situations of uncertainty regarding the diagnosis and the procedure itself (16). The literature highlights that individual vulnerability to stress may be shaped by personality traits. Higher levels of neuroticism are consistently associated with more intense stress experiences, whereas traits such as extraversion, agreeableness and openness appear to serve a protective function, facilitating the use of healthier stress management strategies (17). A growing body of evidence indicates that personality traits and patients’ psychological state play a significant role in the course of treatment for somatic diseases, including cardiovascular disorders. A meta-analysis by Hao Wu et al. found that psychological factors such as anxiety, depression, anger, and occupational stress are significantly associated with an increased risk of atrial fibrillation episodes (18). What is more, a study by Hong Du et al. identified high levels of pre-ablation anxiety as an independent risk factor for atrial fibrillation recurrence (19). As reported by Qian et al. (14), anxiety and depressive symptoms are present in patients with atrial fibrillation, and both conditions may interact, although the underlying mechanism has not yet been fully elucidated. This suggests that patients with atrial fibrillation may be more vulnerable to psychological distress, highlighting the importance of considering psychological factors in the management of atrial fibrillation. Despite these observations, relatively little is known about how stable personality traits contribute to individual differences in psychological responses within these populations. These results highlight the importance of incorporating psychological factors into both risk assessment and the comprehensive management of patients undergoing cardiac care.

Understanding the association of specific personality traits on the level of perceived stress in patients undergoing cardiological interventions has significant clinical and practical implications. Such insight may not only improve the prediction of patients’ emotional responses to planned procedures but also support the development of more personalized psychological support strategies and perioperative care. The aim of the present study is to examine the relationship between selected personality dimensions – Type D personality, the components of the Dark Tetrad, and the subjectively experienced stress levels in patients scheduled for ablation due to atrial fibrillation and atrial flutter.

## Materials and methods

The study protocol was reviewed by the Bioethics Committee (BNW/NWN/0052/KB/293/24). According to the Committee’s decision, the study was exempt from the requirement for full ethical approval due to its non-interventional and questionnaire-based design. It was designed as a single time-point psychological assessment. Data collection was conducted in the Cardiology Department of academic referral hospital between December 5, 2024 and May 5, 2025. The study size was determined by consensus among the authors and the number of eligible patients undergoing planned ablation during the study period. Participants were recruited among patients hospitalized in the cardiology department for a planned ablation procedure. Recruitment took place over multiple days during the study period, while each individual participant was recruited and assessed during a single day. The inclusion criteria were provision of informed consent to participate in the study, a confirmed diagnosis of atrial fibrillation and/or atrial flutter, and age of 18 years or older. The exclusion criteria were current psychotropic treatment or psychotropic treatment received within the preceding two years, the presence of cognitive impairment severe enough to prevent the completion of study questionnaires, and inability to provide informed consent for participation in the study. The main exposure in the study was a confirmed diagnosis of atrial fibrillation and/or atrial flutter. Participants completed a custom-designed questionnaire alongside three standardized psychometric tools mentioned below (Perceived Stress Scale (PSS-10), Short Dark Tetrad Scale (SD4), Type D Personality Scale (DS14)). The custom-designed questionnaire consisted of 20 items assessing demographic characteristics and clinical variables related to atrial fibrillation or flutter. The questionnaire included questions on age, sex, education, place of residence, and family status, as well as clinical variables such as disease duration, symptom severity (EHRA scale), frequency of arrhythmia episodes, stroke history, and circumstances of diagnosis. A substantial part of the questionnaire focused on patients’ health-related behaviors and treatment management, including adherence to medical recommendations, medication-taking behavior, and attitudes toward anticoagulant therapy. It also included items concerning basic mental health history. Most items were closed-ended, with response formats including dichotomous (yes/no) options, single-choice questions, and ordinal rating scales with Likert-type response options. The questionnaire is provided in the **Supplementary Materials**. To reduce the risk of bias, all participants were assessed using the same protocol and data collection methods. Validated assessment tools were applied where applicable, and data collection was performed under consistent clinical conditions. To minimize observer-related variability, all questionnaires were self-administered. This study is reported in accordance with the STROBE guidelines for cross-sectional studies (20).

### 2.1 Perceived stress scale

Perceived Stress Scale (PSS-10), developed by Cohen et al. in 1983, is a self-report tool designed to assess perceived stress in adults (21). It consists of 10 items rated on a 5-point Likert scale. The total score is calculated by summing the item responses, yielding a possible range from 0 to 40. In this study, the Polish adaptation of the scale was used (22).

### 2.2 Short dark tetrad scale

The Short Dark Tetrad Scale (SD4) is a self-report questionnaire designed to measure four subclinical personality traits: Machiavellianism, narcissism, psychopathy, and sadism. Originally developed in 2021 by four researchers (23), it was subsequently translated and psychometrically validated by Dębski et al. into Polish. The Polish version demonstrates satisfactory psychometric characteristics, and the results of its adaptation are currently under review for publication. The scale comprises 28 items scored on a 5-point Likert scale ranging from 1 (strongly disagree) to 5 (strongly agree). Each trait is represented by a 7-item subscale, allowing for the calculation of separate scores.

### 2.3 Type D personality scale

The Type D Personality Scale (DS14) is a self-report measure developed by Denollet in 2005 (24). It evaluates Type D personality traits through 14 items divided equally into two subscales. Each item is rated on a 5-point Likert scale from 0 (false) to 4 (true). Scores are derived by summing responses of each subscale, accounting for reverse rating of two items. The Polish adaptation of DS14 was applied in this research (25).

### 2.4 Statistical analyses

Statistical analyses were performed using Excel 365 and Statistica 13.3 software. The normality of data distribution was assessed based on skewness and kurtosis values. A correlation matrix based on Pearson correlation coefficients was generated for the entire study population, while Spearman’s rank-order correlations were computed separately for female and male subgroups. Group comparisons were conducted using the Mann-Whitney U test. Subsequently, a linear regression model was developed and calculated. Variables included in the regression model were selected based on statistically significant correlations with perceived stress identified in the preliminary analyses. All statistical procedures were carried out with a significance level set at α=0.05.

## Results

### 3.1 Study group characteristics

A total of 50 patients participated in the study, including 21 women (42%) and 29 men (58%). The detailed socio-demographic structure of the study group is presented in **Table 1**. Due to the relatively small sample size, the assessment of the normality of the distribution for the analyses variables was based on skewness and kurtosis values, which are more reliable in small samples. A variable was considered normally distributed if the absolute values of skewness and kurtosis did not exceed 2 (26). This analysis is presented in detail in **Supplementary Tables 1**–**S3** included in the **Supplementary Materials**.

**Table 1:** Socio-demographic structure.

Variable	Mean (SD) or % (n) (N = 50)
Age (years)	60.72 (15.18)
Male age (years)	60.72 (12.03)
Female age (years)	60.71 (19.02)
Sex	
Male	29 (58%)
Female	21 (42%)
Place of residence	
Village	13 (26%)
City <50,000	8 (16%)
City 51,000-150,000	15 (30%)
City 151,000-300,000	11 (22%)
City >300,000	3 (6%)
Children	
Yes	41 (82%)
No	9 (18%)
Close relationship	
Yes	43 (86%)
No	7 (14%)
Employment	
Employed	18 (36%)
Pensioner	32 (64%)
Educational level	
Secondary	21 (42%)
Vocational	13 (26%)
High	16 (32%)
Total years of education	14.48 (3,63)

SD, standard deviation.

### 3.2 Correlation with PSS-10 scores

Within the entire study sample, positive correlations were observed between PSS-10 scores and the levels of psychopathy, social inhibition, and negative affectivity. The full results of these analyses are presented in **Table 2**. Correlation analyses were also conducted separately for male and female participants. However, the results did not differ significantly from those observed in the overall sample. These findings are presented in **Supplementary Tables 4**, **S5** in the **Supplementary Materials**.

**Table 2:** The results of correlation analyses for the study group (p<0.05).

Variable	Age	Total years of education	PSS-10	SD4				DS14	
				Machiavellianism	Narcissism	Psychopathy	Sadism	NA	SI
Age	1								
Total years of education	-0.102803	1							
PSS-10	-0.180223	0.002609	1						
SD4									
Machiavellianism	0.049769	-0.193797	0.117177	1					
Narcissism	-0.114593	-0.066920	0.214556	0.353100*	1				
Psychopathy	-0.055608	0.012074	0.308476*	0.323319*	0.515390*	1			
Sadism	-0.171210	0.012921	0.153484	0.194274	0.321253*	0.662035*	1		
DS14									
NA	0.035546	-0.066626	0.735184*	0.115544	0.142154	0.233641	0.044418	1	
SI	0.126074	0.030422	0.336871*	-0.011995	-0.115656	-0.010895	0.011331	0.534497*	1

PSS-10, Perceived Stress Scale; SD4, Short Dark Tetrad Scale; DS14, Type D Personality Scale; NA, negative affectivity; SI, social inhibition.

*statistically significant result.

### 3.3 Comparisons – sex and selected health parameters

The greatest number of statistically significant differences within the study group emerged after dividing the participants by sex. Sadism intensity was significantly higher among men, whereas women exhibited significantly higher levels of stress symptoms, negative affectivity, and social inhibition. All analyses are presented in **Table 3**.

**Table 3:** The results of the comparative analyses for the groups of women and men (p<0.05).

Variable	Men (n=29)		Women (n=21)				Z	p
	Mean	SD	± 95% CI for Mean	Mean	SD	± 95% CI for Mean		
PSS-10	12.48	6.19	10.13-14.83	18.52	8.08	14.84-22.20	-2.58	0.009856
Machiavellianism	21.03	6.44	18.58-23.48	22.33	3.92	20.54-24.12	-1.08	0.278225
Narcissism	18.59	5.84	16.36-20.82	16.90	4.90	14.67-19.13	1.50	0.133494
Psychopathy	13.90	5.51	11.79-16.01	12.62	5.05	10.32-14.92	0.80	0.424676
Sadism	13.90	5.18	11.91-15.89	10.76	4.93	8.52-13.00	2.42	0.015481
NA	8.34	6.52	5.86-10.82	13.62	6.29	10.76-16.48	-2.54	0.011073
SI	7.34	4.65	5.57-9.11	10.86	4.51	8.81-12.91	-2.55	0.010928

SD, standard deviation; PSS-10, Perceived Stress Scale; SD4, Short Dark Tetrad Scale; DS14, Type D Personality Scale; NA, negative affectivity; SI, social inhibition.

**Figure 1:**
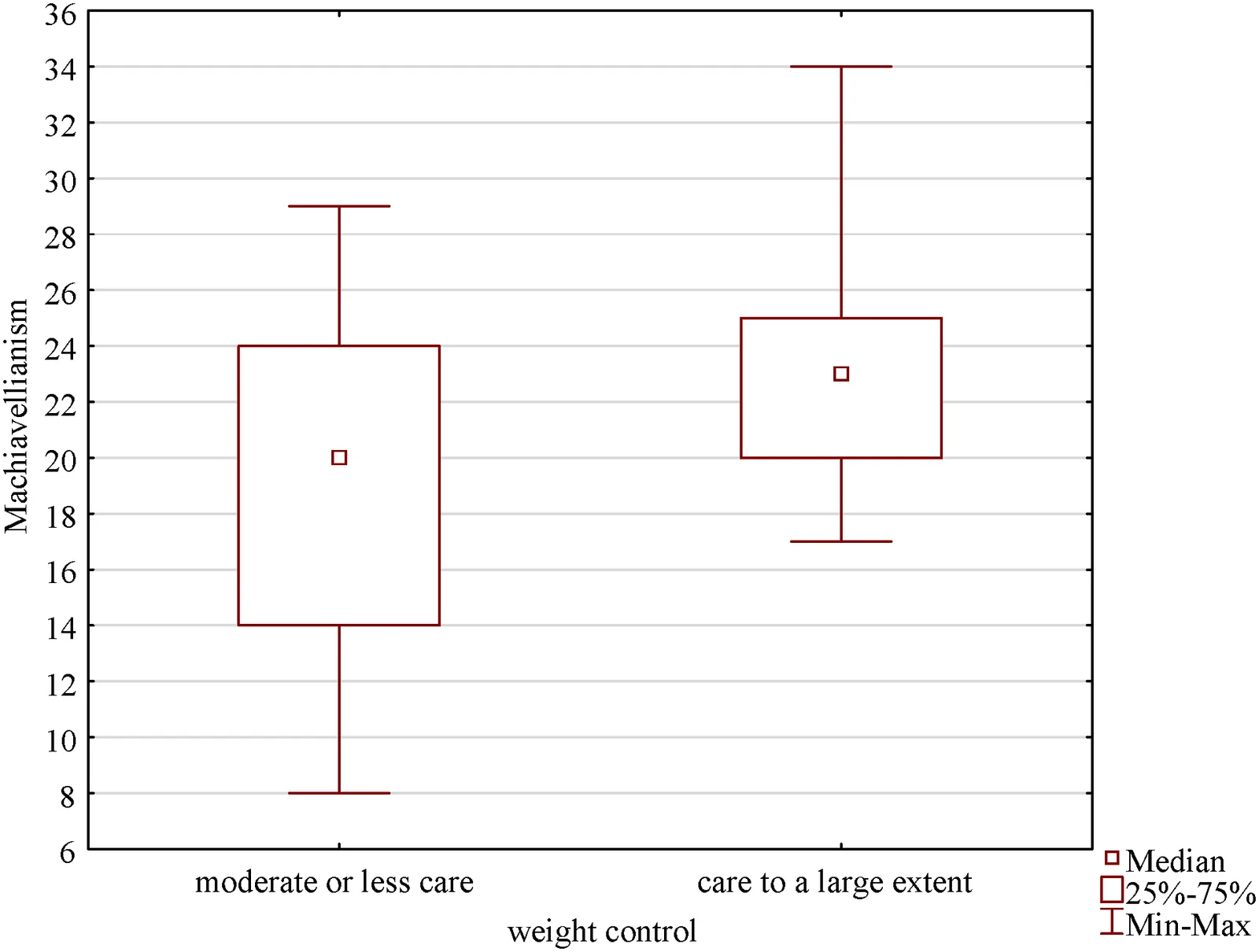
Comparison of Machiavellianism scores according to the degree of weight control care (p<0.05).

**Figure 2:**
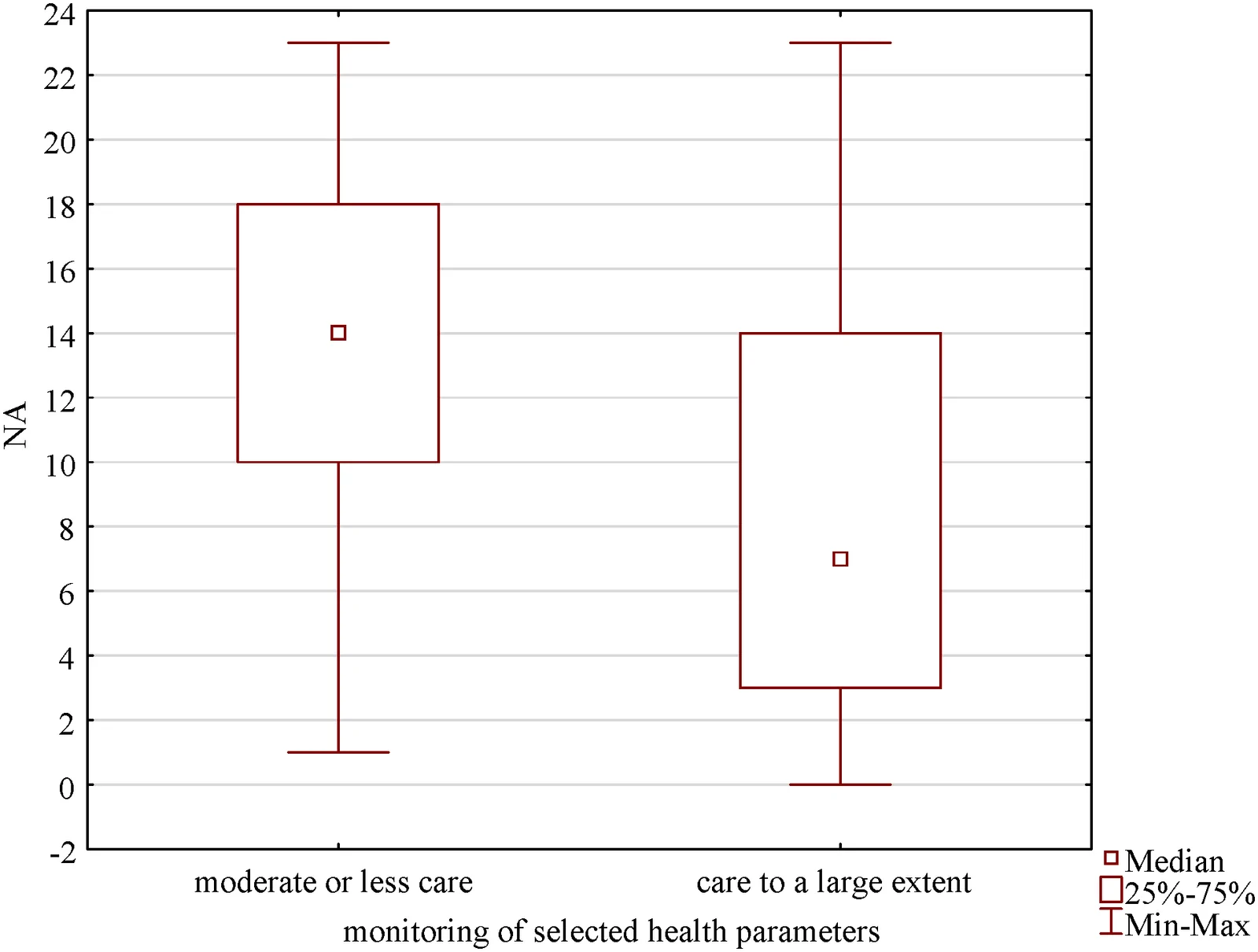
Comparison of negative affectivity scores according to the degree of monitoring selected health parameters (p<0.05).

**Figure 3:**
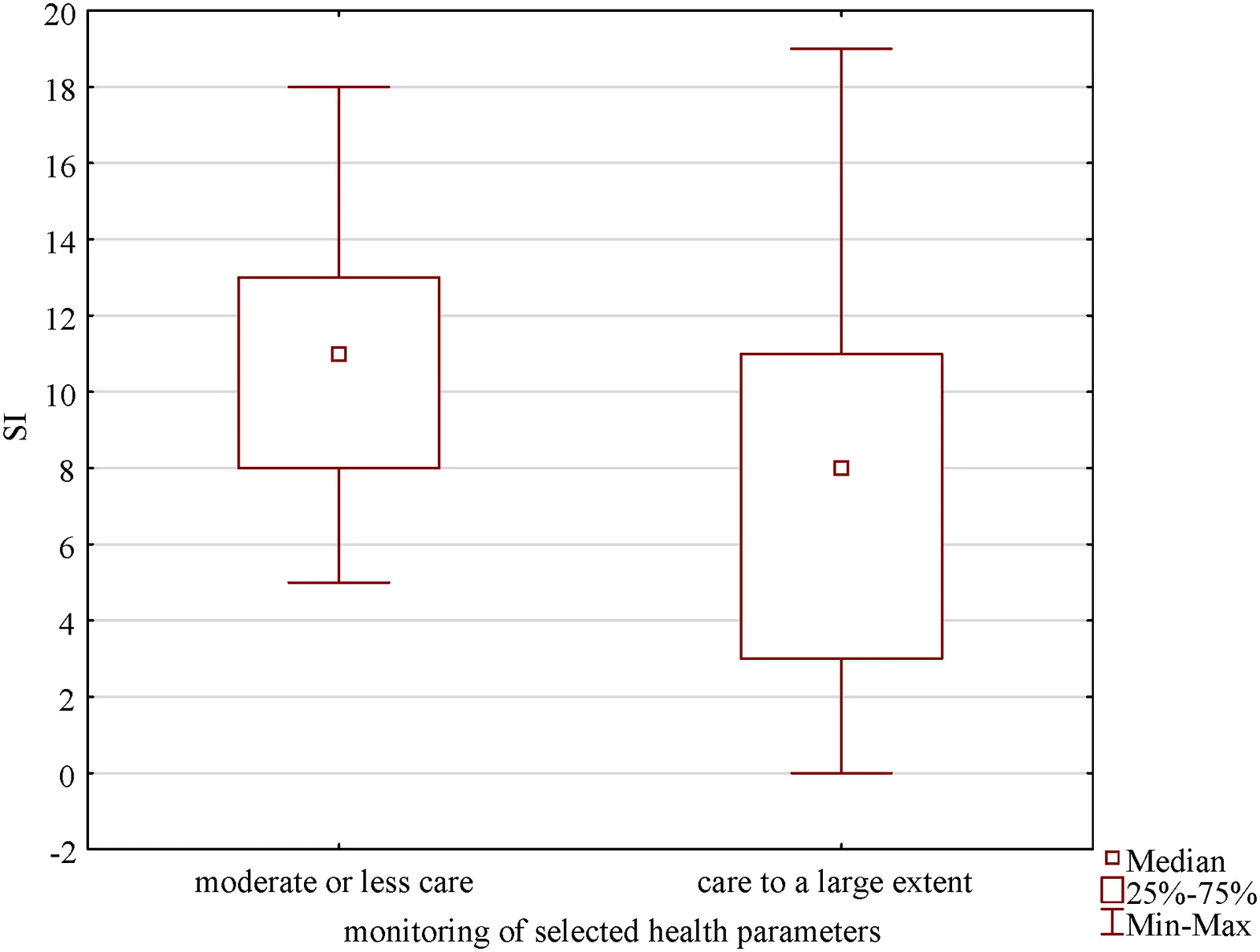
Comparison of social inhibition scores according to the degree of monitoring selected health parameters (p<0.05).

Among the other variables, a statistically significant difference was observed in the level of Machiavellianism, which was higher among individuals who reported greater concern with maintaining a healthy body weight. This finding is illustrated in **Figure 1**. Further analyses focused on involvement in monitoring selected health parameters (such as blood pressure, blood glucose levels, the presence of obstructive sleep apnea, and cholesterol levels). For these analyses, responses were dichotomized into two categories: high involvement (“to a great extent”) and moderate or lower involvement (“to a moderate extent”, “to a slight extent”, “to a minimal extent”, or “not at all”). Participants reporting lower engagement in managing these factors demonstrated significantly higher levels of negative affectivity and social inhibition. These findings are depicted in **Figures 2**, **3**.

### 3.4 Perceived stress level regression analysis

A linear regression model was constructed to examine the potential association between selected personality traits and perceived stress level. Variables included in the regression model were selected based on statistically significant correlations with perceived stress identified in the preliminary analyses. The results are presented in **Table 4**. Before conducting the regression analyses, the assumptions of linear regression were examined. The normality of the residual distribution was assessed using Q–Q plots, which showed no significant deviations from normality (**Supplementary Figure S1**). Homoscedasticity and linearity were assessed by visual inspection of the residuals against the fitted values, revealing no clear violations (**Supplementary Figure S2**). Potential outliers were examined using Cook’s distance, and no cases with a significant impact were identified (**Supplementary Figure S3**). Given the relatively small sample size, minor deviations were deemed acceptable and did not affect the interpretation of the results.

**Table 4:** The regression analyses results for perceived stress level as the dependent variable (p<0.05).

Variable	b	b SE	± 95% CI for b	β	β SE	± 95% CI for Beta	t	p	Statistics
Constant term	4.59	2.43	-0.30-9.48	–	–	–	1.89	0.064781	R^2^ = 0.53, F(3.46)=19.69, p<0.001, SEE = 5.19
Psychopathy	0.14	0.10	-0.06-0.34	0.20	0.15	-0.10-0.50	1.35	0.184487	
NA	0.73	0.12	0.49-0.97	0.81	0.13	0.55-1.07	6.07	<0.001	
SI	-0.05	0.12	-0.29-0.19	-0.08	0.18	-0.44-0.28	-0.45	0.656097	

SE, standard error; SEE, standard error of estimate; NA, negative affectivity; SI, social inhibition.

## Discussion

As a result of our analyses, positive correlations were identified between PSS-10 scores and levels of psychopathy, social inhibition and negative affectivity (components of Type D personality) across patients undergoing catheter ablation for atrial fibrillation or atrial flutter. These findings suggest that individuals with stronger expression of these traits tend to experience greater levels of perceived stress. The observed association between psychopathy and perceived stress appears somewhat inconsistent with the findings of Paulhus and Williams (5), who reported that psychopathy, unlike the other components of the Dark Triad (narcissism and Machiavellianism), is characterized by relatively low levels of neuroticism. Given that low neuroticism may be associated with fewer negative emotions and lower vulnerability to stress (27), psychopathy would be expected to be associated with lower rather than higher levels of perceived stress. However, psychopathic traits have also been linked to impulsivity and low frustration tolerance, which may increase individuals’ reactivity to stressful or frustrating situations and thereby contribute to higher levels of perceived stress (28).

Importantly, in the regression model, negative affectivity remained the only significant predictor of perceived stress. This finding is consistent with O’Riordan et al. (2020), who reported that Type D individuals tend to perceive stressful events as more stressful than non-Type D individuals, and further demonstrated that this effect is primarily driven by the NA subcomponent. The authors also noted that their findings are consistent with previous research (29).

Regarding the correlations between stress levels and Dark Triad traits, the present study did not identify associations for Machiavellianism or narcissism. However, in the study by Wertag et al. (30), Machiavellianism and narcissism were found to be positively and negatively correlated with stress, respectively. With respect to psychopathy, sadism, and stress levels, the authors reported non-significant associations. Moreover, they noted that findings regarding the relationship between psychopathy and stress remain inconsistent. It is important to note that each of the mentioned studies was conducted under conditions of participants’ normal daily functioning. Our study was the only one carried out during hospitalization and assumed the presence of a stressor, in the form of an upcoming medical procedure. These factors may have contributed to the uniqueness of our findings. It is also worth noting that the highest levels of stress were observed among patients residing in large urban areas. This may be related to additional environmental burdens associated with urban lifestyles or to greater access to healthcare services and information, which may, in turn, lead to increased awareness of one’s health status, illness perception, and potential complications related to the procedure.

An interesting finding of the study is that individuals who reported maintaining a healthy body weight exhibited Machiavellian traits more frequently. This behavior may be interpreted as an example of a health-promoting attitude. However, this finding stands in contrast to the results of previous research, which has shown that individuals with high levels of Machiavellianism are more likely to engage in health-damaging behaviors and display antisocial tendencies (31, 32). On the other hand, individuals high in Machiavellianism are typically strongly goal-oriented, and improving one’s health by maintaining a healthy weight may represent such a goal. Conversely, individuals who reported less concern with maintaining these health parameters were characterized by higher levels of negative affectivity and social inhibition (component of Type D personality). Such neglect of physical health may be associated with maladaptive emotional and social functioning. Another possible explanation for this association may involve elevated depressive and anxiety symptoms frequently observed in individuals with Type D personality (4). However, these variables were not directly assessed in the present study.

Statistical analysis revealed that individuals who reported lower levels of engagement in monitoring blood pressure, blood glucose level, obstructive sleep apnea, and cholesterol levels scored significantly higher on both subscales of the DS14 questionnaire. Similar results were reported by Kim et al. (33) among post-stroke patients, as well as by Buczkowska et al. (34) in patients with obesity. Buczkowska’s study also demonstrated that Type D personality in patients with cardiovascular disease was associated with lower subjective health evaluations, reduced engagement in the treatment process, and more frequent somatic complaints, such as headaches and sleep disturbances. Likewise, Basińska et al. (35) found that patients with Type D personality were less likely to engage in behaviors conducive to recovery and that their health-related attitudes were characterized by an external locus of control. Based on the reviewed studies, it appears that the observed correlation is relatively common across a range of somatic conditions.

Gender differences revealed significantly higher levels of sadism among men, and higher levels of stress, negative affectivity, and social inhibition among women. These findings are consistent with previous literature (36, 37) suggesting that the examined sample of patients with atrial fibrillation and flutter did not significantly differ from populations studied in earlier research in terms of the observed psychological characteristics.

The findings of our study contribute to a topic that remains relatively underexplored in contemporary research. The existing literature contains few studies addressing similar issues in cardiology patients. Most available studies have focused primarily on healthy populations (28, 30, 38, 39). Among studies on related topics, several have examined the associations between traits of the “Dark Triad” and health-related behaviors (31, 32, 40), as well as the severity of anxiety and depressive symptoms (41, 42). Studies examining correlations between Type D personality and the intensity and nature of atrial fibrillation symptoms can be also found in the literature (43, 44). It is worth noting that most available publications concerning the “dark core of personality” focus primarily on the Dark Triad. In contrast, the present study focuses on the “Dark Tetrad”, which also includes sadism. Moreover, there are few studies addressing atrial flutter.

This study was conducted with methodological rigor, utilizing appropriate instruments and procedures. A comprehensive statistical analysis was carried out, and the obtained results were thoroughly discussed and compared with the existing literature. Despite these efforts, several important limitations must be acknowledged. Most notably, the relatively small sample size (n=50) substantially limits statistical power and reduces the generalizability of the findings. Therefore, the present results should be interpreted as preliminary and exploratory. Furthermore, the cross-sectional design of the study does not allow for conclusion regarding causality between the investigated variables. Another limitation was the use of relatively non-restrictive inclusion and exclusion criteria, which resulted in heterogeneous patients’ population. Tightening the eligibility criteria could have reduced the impact of potential confounding factors, such as differences in demographic characteristics, comorbidity burden or pharmacological treatment, which could have influenced both perceived stress and personality assessment outcomes. This approach might have increased the validity of the results and enabled a more accurate assessment of the emotional response specifically related to the medical procedure. Another limitation concerns the regression analysis conducted in a relatively small sample, which may limit the stability and robustness of the obtained model. The study also does not include a control group, which limits the ability to compare the results with a reference population of individuals without atrial fibrillation or atrial flutter or with a cohort of patients with these arrhythmias who are managed outside of the hospital setting. Including such a control group would have expanded the study’s scope and allowed for the assessment of additional factors potentially influencing the observed outcomes. Although the present study highlights the relevance of psychological traits in patients with atrial fibrillation and atrial flutter, no subgroup analyses were conducted comparing patients with different arrhythmia types. Future research should address these relationships, as they may provide further insight into the interaction between clinical presentation and psychological functioning in this patient population. Nevertheless, a key strength of our study lies in its attempt to explore selected aspects of psychological functioning in the context of personality traits within a specific cardiology patient group - individuals undergoing ablation for atrial fibrillation of flutter. Future studies could also consider incorporating objective measures of body weight, as well as patients’ subjective evaluation and their level of acceptance of body image. The limitations described above provide valuable guidance for future research on similar topics. Therefore, the present findings should be considered preliminary and hypothesis-generating rather than conclusive.

## Conclusion

The present study identified potential associations between selected Dark Tetrad traits, Type D personality dimensions, and perceived stress in patients undergoing ablation for atrial fibrillation or atrial flutter. Due to the exploratory and cross-sectional nature of the study, as well as the relatively small sample size, the findings should be interpreted cautiously. Nevertheless, the results suggest that psychological characteristics may be relevant to patients’ perioperative experiences and may support the future development of more individualized psychological care strategies. Further studies involving larger samples, control groups, and longitudinal follow-up are required.

## Data Availability

The raw data supporting the conclusions of this article will be made available by the authors, without undue reservation.

## Supplementary material

Supplementary Figure 1 Normal probability plot.

Supplementary Figure 2 Residuals versus predicted.

Supplementary Figure 3 Cook’s distance.

## References

[1] Rozanski A Blumenthal JA Kaplan J . Impact of psychological factors on the pathogenesis of cardiovascular disease and implications for therapy. Circulation. (1999) 99:2192–217. doi: 10.1161/01.cir.99.16.2192 10217662

[2] Friedman M Rosenman RH . Association of specific overt behavior pattern with blood and cardiovascular findings. JAMA. (1959) 169:1286–96. doi: 10.1001/jama.1959.03000290012005 13630753

[3] Denollet J Rombouts H Gillebert TC Brutsaert DL Sys SU Stroobant N . Personality as independent predictor of long-term mortality in patients with coronary heart disease. Lancet. (1996) 347:417–21. doi: 10.1016/s0140-6736(96)90007-0 8618481

[4] Kupper N Denollet J . Type D personality as a risk factor in coronary heart disease: a review of current evidence. Curr Cardiol Rep. (2018) 20:104. doi: 10.1007/s11886-018-1048-x 30209683 PMC6153564

[5] Paulhus DL Williams KM . The dark triad of personality: narcissism, Machiavellianism and psychopathy. J Res Pers. (2002) 36:556–63. doi: 10.1016/S0092-6566(02)00505-6

[6] Buckels EE Jones DN Paulhus DL . Behavioral confirmation of everyday sadism. Psychol Sci. (2013) 24:2201–9. doi: 10.1177/0956797613490749 24022650

[7] Hilbig BE Thielmann I Klein SA Moshagen M Zettler I . The dark core of personality and socially aversive psychopathology. J Pers. (2021) 89:216–27. doi: 10.1111/jopy.12577 32654193

[8] Christie R Geis FL . Studies in Machiavellianism. New York: Academic Press (1970).

[9] Mitra P Torrico TJ Fluyau D . Narcissistic personality disorder. In: Statpearls. StatPearls Publishing, Treasure Island (FL (2024). 32310461

[10] Fisher KA Torrico TJ Hany M . Antisocial personality disorder. In: Statpearls. StatPearls Publishing, Treasure Island (FL (2024).

[11] Porter S Bhanwer A Woodworth M Black PJ . Soldiers of misfortune: An examination of the Dark Triad and the experience of sChadenfreude. Pers Individ Dif. (2014) 67:64–8. doi: 10.1016/j.paid.2013.11.014 38826717

[12] Kalarus Z Średniawa B Mitręga K Wierucki Ł Sokal A Lip G . Prevalence of atrial fibrillation in the 65 or over Polish population. Report of cross-sectional NOMED-AF study. Pol Heart J (Kardiol Pol). (2023) 81:14–21. doi: 10.33963/KP.a2022.0202 36043418

[13] Hindricks G Potpara T Dagres N Arbelo E Bax JJ Blomström-Lundqvist C . 2020 ESC guidelines for the diagnosis and management of atrial fibrillation developed in collaboration with the European Association for Cardio-Thoracic Surgery (EACTS). Eur Heart J. (2021) 42:373–498. doi: 10.1093/eurheartj/ehaa612 32860505

[14] Qian L Shen Y . Anxiety and depression in patients undergoing catheter ablation due to atrial fibrillation: A cross-sectional survey. Heliyon. (2024) 10:e39788. doi: 10.1016/j.heliyon.2024.e39788 39687192 PMC11647955

[15] Rosiek A Kornatowski T Rosiek-Kryszewska A Leksowski Ł Leksowski K . Evaluation of stress intensity and anxiety level in preoperative period of cardiac patients. BioMed Res Int. (2016) 2016:1248396. doi: 10.1155/2016/1248396 27042655 PMC4793098

[16] Piegza M Pudlo R Badura-Brzoza K Piegza J Szyguła-Jurkiewicz B Gorczyca P . Dynamics of anxiety in woman undergoing coronary angiography. Pol Heart J (Kardiol Pol). (2014) 72:175–80. doi: 10.5603/KP.a2013.0214 23990234

[17] Luo J Zhang B Cao M Roberts BW . The stressful personality: A meta-analytical review of the relation between personality and stress. Pers Soc Psychol Rev. (2023) 27:128–94. doi: 10.1177/10888683221104002 35801622

[18] Wu H Li C Li B Zheng T Feng K Wu Y . Psychological factors and risk of atrial fibrillation: A meta-analysis and systematic review. Int J Cardiol. (2022) 362:85–92. doi: 10.1016/j.ijcard.2022.05.048 35618103

[19] Du H Yang L Hu Z Zhang H . Anxiety is associated with higher recurrence of atrial fibrillation after catheter ablation: A meta-analysis. Clin Cardiol. (2022) 45:243–50. doi: 10.1002/clc.23753 35043425 PMC8922539

[20] von Elm E Altman DG Egger M Pocock SJ Gøtzsche PC Vandenbroucke JP . The Strengthening the Reporting of Observational Studies in Epidemiology (STROBE) statement: guidelines for reporting observational studies. J Clin Epidemiol. (2008) 61:344–9. doi: 10.1016/j.jclinepi.2007.11.008 18313558

[21] Cohen S Kamarck T Mermelstein R . A global measure of perceived stress. J Health Soc Behav. (1983) 24:385–96. doi: 10.2307/2136404 6668417

[22] Juczyński Z Ogińska-Bulik N . Narzędzia Pomiaru Stresu I Radzenia Sobie Ze Stresem. Warszawa: Pracownia Testów Psychologicznych Polskiego Towarzystwa Psychologicznego (2009).

[23] Paulhus DL Buckels EE Trapnell PD Jones DN . Screening for Dark Personalities: The Short Dark Tetrad (SD4). Eur J Psychol Assess. (2021) 37(3):208–222. doi: 10.1027/1015-5759/a000602

[24] Denollet J . DS14: Standard assessment of negative affectivity, social inhibition, and Type D personality. Psychosom Med. (2005) 67:89–97. doi: 10.1097/01.psy.0000149256.81953.49 15673629

[25] Poprawa R Juczyński Z . Skala typu D (DS14). In: Juczyński Z Ogińska-Bulik N , editors. Narzędzia Pomiaru Stresu I Radzenia Sobie Ze Stresem. Pracownia Testów Psychologicznych PTP, Warszawa (2009). p. 59–68.

[26] George D Mallery P . IBM SPSS Statistics 27 Step by Step: A Simple Guide and Reference. New York: Routledge (2021).

[27] Tananuvat N Tansanguan S Wongpakaran N Wongpakaran T . Role of neuroticism and perceived stress on quality of life among patients with dry eye disease. Sci Rep. (2022) 12:7198. doi: 10.1038/s41598-022-11271-z 35490178 PMC9056508

[28] Noser AE Zeigler-Hill V Besser A . Stress and affective experiences: The importance of dark personality features. J Res Pers. (2014) 53:158–64. doi: 10.1016/j.jrp.2014.10.007 38826717

[29] O’Riordan A Howard S Gallagher S . Type D personality and life event stress: the mediating effects of social support and negative social relationships. Anxiety Stress Coping. (2020) 33:452–65. doi: 10.1080/10615806.2020.1746284 32223435

[30] Wertag A Milas G Ribar M . A cross-lagged relationship of subjective stress and dark tetrad traits in adolescence. Pers Individ Dif. (2025) 240:113137. doi: 10.1016/j.paid.2025.113137 38826717

[31] Dębska M Dębski P Polechoński J Rozpara M Tomik R . The Dark Triad of personality in the context of health behaviors: Ally or enemy? Int J Environ Res Public Health. (2021) 18:4113. doi: 10.3390/ijerph18084113 33924649 PMC8069531

[32] Jonason PK Baughman HM Carter GL Parker P . Dorian Gray without his portrait: Psychological, social, and physical health costs associated with the Dark Triad. Pers Individ Dif. (2015) 78:5–13. doi: 10.1016/j.paid.2015.01.008 38826717

[33] Kim SR Kim S Cho BH Yu S Cho KH . Influence of Type D personality on health promoting behaviours and quality of life in stroke patients: A cross-sectional study in South Korea. J Stroke Cerebrovasc Dis. (2021) 30:105721. doi: 10.1016/j.jstrokecerebrovasdis.2021.105721 33735669

[34] Buczkowska M Górski M Domagalska J Buczkowski K Nowak P . Type D personality and health behaviors in people living with obesity. Int J Environ Res Public Health. (2022) 19:14650. doi: 10.3390/ijerph192214650 36429364 PMC9690440

[35] Basińska M Andruszkiewicz A . Cechy osobowości typu D a funkcjonowanie w chorobie pacjentów ze schorzeniami przewlekłymi. Pol Forum Psychol. (2016) 21:221–37. doi: 10.14656/PFP20160205

[36] McLean CP Asnaani A Litz BT Hofmann SG . Gender differences in anxiety disorders: prevalence, course of illness, comorbidity and burden of illness. J Psychiatr Res. (2011) 45:1027–35. doi: 10.1016/j.jpsychires.2011.03.006 21439576 PMC3135672

[37] Cale EM Lilienfeld SO . Sex differences in psychopathy and antisocial personality disorder. A review and integration. Clin Psychol Rev. (2002) 22:1179–207. doi: 10.1016/s0272-7358(01)00125-8 12436810

[38] Domagalska J Rusin M Razzaghi M Nowak P . Personality Type D, level of perceived stress, insomnia, and depression among high school teachers in Poland. Front Psychol. (2021) 12:626945. doi: 10.3389/fpsyg.2021.626945 34621203 PMC8490815

[39] Sumin A Zagorskaya N Shcheglova A Shipilov A Kostylbaev D Shikanova E . Personality Type D and psychophysiological stress reactivity during mental stress in young healthy individuals. Behav Sci (Basel). (2025) 15:852. doi: 10.3390/bs15070852 40723636 PMC12292575

[40] Malesza M Kaczmarek MC . Dark side of health-predicting health behaviors and diseases with the Dark Triad traits. J Public Health. (2021) 29:275–84. doi: 10.1007/s10389-019-01129-6 30311153

[41] Li J Liu C Albertella L Rotaru K Li K Yu Z . Network analysis of the association between Dark Triad traits and depression symptoms in university students. Pers Individ Dif. (2024) 218:112495–115. doi: 10.1016/j.paid.2023.112495 38826717

[42] Gómez-Leal R Megías-Robles A Gutiérrez-Cobo MJ Cabello R Fernández-Abascal EG Fernández-Berrocal P . Relationship between the Dark Triad and depressive symptoms. PeerJ. (2019) 7:e8120. doi: 10.7717/peerj.8120 31803535 PMC6886484

[43] Kupper N van den Broek K Haagh E van der Voort P Widdershoven J Denollet J . Type D personality affects health-related quality of life in patients with lone atrial fibrillation by increasing symptoms related to sympathetic activation. J Psychosom Res. (2018) 115:44–52. doi: 10.1016/j.jpsychores.2018.10.005 30470316

[44] Thompson TS Barksdale DJ Sears SF Mounsey JP Pursell I Gehi AK . The effect of anxiety and depression on symptoms attributed to atrial fibrillation. Pacing Clin Electrophysiol. (2014) 37:439–46. doi: 10.1111/pace.12292 24215267

