# Supplementary material for: An analysis of the relationship of selected personality traits on perceived stress in patients undergoing ablation for atrial fibrillation and atrial flutter
Source: Front Psychiatry. 2026 Aug 10;17:1681576. doi: 10.3389/fpsyt.2026.1681576 (PMC13501273; doi:10.3389/fpsyt.2026.1681576)
Supplement: Supplementary file 1 [file Table1.docx]

Supplementary Material

TABLE S1 Assesment of the normal distribution in research group.

| **Variable** | **Mean (SD)** | **Minimum** | **Maximum** | **Skewness** | **Kurtosis** |
| --- | --- | --- | --- | --- | --- |
| Age (years) | 60.72 (15.18) | 24 | 84 | -0.77 | -0.20 |
| Total years of education | 14.48 (3.63) | 9 | 26 | 1.13 | 1.52 |
| PSS-10 | 15.02 (7.59) | 0 | 34 | 0.46 | 0.31 |
| SD4 | | | | | |
| Machiavellianism | 21.58 (5.51) | 8 | 34 | -0.11 | 0.25 |
| Narcissism | 17.88 (5.48) | 7 | 29 | -0.14 | -0.24 |
| Psychopathy | 13.36 (5.29) | 7 | 28 | 0.94 | 0.38 |
| Sadism | 12.58 (5.26) | 7 | 28 | 1.04 | 0.98 |
| DS14 | | | | | |
| NA | 10.56 (6.88) | 0 | 23 | 0.03 | -1.07 |
| SI | 8.82 (4.87) | 0 | 19 | 0.23 | -0.57 |

*SD, standard deviation; PSS-10, Perceived Stress Scale; SD4, Short Dark Tetrad Scale; DS14, Type D Personality Scale; NA, negative affectivity; SI, social inhibition.*

TABLE S2 Assesment of the normal distribution in women

| **Variable** | **Mean (SD)** | **Minimum** | **Maximum** | **Skewness** | **Kurtosis** |
| --- | --- | --- | --- | --- | --- |
| Age (years) | 60.71 (19.02) | 24 | 84 | -0.83 | -0.54 |
| Total years of education | 14.76 (2.61) | 11 | 18 | 0.03 | -1.68 |
| PSS-10 | 18.52 (8.08) | 5 | 34 | 0.36 | -0.35 |
| SD4 | | | | | |
| Machiavellianism | 22.33 (3.92) | 13 | 29 | -0.25 | 0.37 |
| Narcissism | 16.90 (4.90) | 8 | 29 | 0.46 | 0.97 |
| Psychopathy | 12.62 (5.02) | 7 | 28 | 1.61 | 3.35 |
| Sadism | 10.76 (4.93) | 7 | 27 | 1.96 | 4.91 |
| DS14 | | | | | |
| NA | 13.62 (6.29) | 0 | 23 | -0.35 | -0.23 |
| SI | 10.86 (4.51) | 2 | 19 | 0.01 | -0.41 |

*SD, standard deviation; PSS-10, Perceived Stress Scale; SD4, Short Dark Tetrad Scale; DS14, Type D Personality Scale; NA, negative affectivity; SI, social inhibition.*

TABLE S3 Assesment of the normal distribution in men.

| **Variable** | **Mean (SD)** | **Minimum** | **Maximum** | **Skewness** | **Kurtosis** |
| --- | --- | --- | --- | --- | --- |
| Age (years) | 60.72 (12.03) | 37 | 76 | -0.51 | -0.95 |
| Total years of education | 14.28 (4.25) | 9 | 26 | 1.36 | 1.54 |
| PSS-10 | 12.48 (6.19) | 0 | 26 | -0.01 | 0.04 |
| SD4 | | | | | |
| Machiavellianism | 21.03 (6.44) | 8 | 34 | 0.08 | -0.18 |
| Narcissism | 18.59 (5.84) | 7 | 29 | -0.52 | -0.19 |
| Psychopathy | 13.90 (5.51) | 7 | 25 | 0.60 | -0.49 |
| Sadism | 13.90 (5.18) | 7 | 28 | 0.72 | 0.61 |
| DS14 | | | | | |
| NA | 8.34 (6.52) | 0 | 20 | 0.32 | -1.20 |
| SI | 7.34 (4.65) | 0 | 19 | 0.51 | -0.10 |

*SD, standard deviation; PSS-10, Perceived Stress Scale; SD4, Short Dark Tetrad Scale; DS14, Type D Personality Scale; NA, negative affectivity; SI, social inhibition.*

TABLE S4 The results of correlation analyses for women (p<0.05).

| **Variable** | **Age** | **Total years of education** | **PSS-10** | **SD4** | | | | **DS14** | |
| --- | --- | --- | --- | --- | --- | --- | --- | --- | --- |
|  |  |  |  | **Machiavellianism** | **Narcissism** | **Psychopathy** | **Sadism** | **NA** | **SI** |
| Age | 1 |  |  |  |  |  |  |  |  |
| Total years of education | -0.34 | 1 |  |  |  |  |  |  |  |
| PSS-10 | -0.07 | -0.01 | 1 |  |  |  |  |  |  |
| SD4 | | | | | | | | | |
| Machiavellianism | 0.13 | -0.05 | 0.34 | 1 |  |  |  |  |  |
| Narcissism | -0.06 | 0.07 | 0.28 | 0.16 | 1 |  |  |  |  |
| Psychopathy | 0.26 | -0.16 | 0.36 | 0.40 | 0.30 | 1 |  |  |  |
| Sadism | 0.02 | 0.31 | 0.02 | 0.10 | 0.17 | 0.47* | 1 |  |  |
| DS14 | | | | | | | | | |
| NA | -0.05 | -0.09 | 0.74* | 0.24 | 0.50* | 0.22 | -0.06 | 1 |  |
| SI | 0.12 | -0.19 | 0.32 | -0.12 | 0.23 | 0.18 | -0.06 | 0.54* | 1 |

*PSS-10, Perceived Stress Scale; SD4, Short Dark Tetrad Scale; DS14, Type D Personality Scale; NA, negative affectivity; SI, social inhibition.*

**—statistically significant result*

TABLE S5 The result of correlation analyses for men (p<0.05).

| **Variable** | **Age** | **Total years of education** | **PSS-10** | **SD4** | | | | **DS14** | |
| --- | --- | --- | --- | --- | --- | --- | --- | --- | --- |
|  |  |  |  | **Machiavellianism** | **Narcissism** | **Psychopathy** | **Sadism** | **NA** | **SI** |
| Age | 1 |  |  |  |  |  |  |  |  |
| Total years of education | 0.14 | 1 |  |  |  |  |  |  |  |
| PSS-10 | -0.21 | 0.04 | 1 |  |  |  |  |  |  |
| SD4 | | | | | | | | | |
| Machiavellianism | 0.08 | -0.09 | -0.01 | 1 |  |  |  |  |  |
| Narcissism | -0.21 | -0.03 | 0.28 | 0.38* | 1 |  |  |  |  |
| Psychopathy | -0.13 | 0.07 | 0.38* | 0.36 | 0.60* | 1 |  |  |  |
| Sadism | -0.12 | -0.02 | 0.37* | 0.36 | 0.46* | 0.63* | 1 |  |  |
| DS14 | | | | | | | | | |
| NA | -0.05 | -0.02 | 0.52* | 0.07 | 0.09 | 0.42* | 0.35 | 1 |  |
| SI | 0.08 | 0.21 | 0.08 | -0.03 | -0.25 | -0.11 | 0.13 | 0.46* | 1 |

*PSS-10, Perceived Stress Scale; SD4, Short Dark Tetrad Scale; DS14, Type D Personality Scale; NA, negative affectivity; SI, social inhibition.*

**—statistically significant result*
