# Supplementary material for: An analysis of the relationship of selected personality traits on perceived stress in patients undergoing ablation for atrial fibrillation and atrial flutter
Source: Front Psychiatry. 2026 Aug 10;17:1681576. doi: 10.3389/fpsyt.2026.1681576 (PMC13501273; doi:10.3389/fpsyt.2026.1681576)
Supplement: Supplementary file 2 [file Table2.docx]

Invitation to Participate in a Research Study,

We would like to invite you to participate in a research study entitled “An analysis of the relationship of selected personality traits on perceived stress in patients undergoing ablation for atrial fibrillation and atrial flutter”.

The aim of this study is to examine the relationship between selected personality traits and the level of perceived stress among patients preparing to undergo an ablation procedure. Your participation will contribute to a better understanding of these relationships, which may help improve medical care and psychological support for patients in the future.

Participation in the study involves completing a demographic questionnaire and standardized questionnaires assessing the above-mentioned issues. The study is cross-sectional and consists of a single assessment (you will not be asked to complete any additional questionnaires or provide further responses in the future). Participation in the study is entirely voluntary. You may withdraw from the study at any time without providing a reason and without any negative consequences.

All questionnaires are anonymous, and the data collected will be used solely for scientific purposes and statistical analyses. Access to the data will be restricted to members of the research team.

The study is being conducted by a team of specialists from the Department and Clinical Ward of Psychiatry in Tarnowskie Góry, the First Department of Cardiology and Angiology of the Silesian Center for Heart Diseases, and fifth-year medical students of the Medical University of Silesia who are members of the Student Scientific Society affiliated with the Department of Psychiatry.

The estimated time required to complete the study is approximately 15 minutes.

Thank you for your time and your participation in this study.

1. **Gender:**
   a) Female
   b) Male
   c) Other
2. **Age *(please specify in years)***
   a) …
3. **Place of residence:**
   a) Rural area
   b) City up to 50 000 inhabitants
   c) City 51 000 – 150 000 inhabitants
   d) City 151 000 – 300 000 inhabitants
   e) City over 300 000 inhabitants

4. **Relationship status:**
   a) Single
   b) In a domestic partnership
   c) Married
5. **Do you have any children?**
   a) Yes
   b) No
6. **Employment status (*please select the option that best describes you*):**
   a) Employed
   b) Student/pupil
   c) Retired and/or pensioner
   d) Unemployed
7. **Education level:**
   a) Primary or lower secondary
   b) Vocational
   c) Secondary
   d) Higher
8. **Total years of education *(please specify in years)***

**- …**

9. **How long have you been treated for atrial fibrillation or atrial flutter**
   a) New diagnosis (during current hospitalization)
   b) For several weeks
   c) For several months
   d) For one year
   e) For several years
10. **Under what circumstances was your atrial fibrillation or atrial flutter diagnosed?**
    a) Symptoms (e.g., palpitations, paroxysmal sweating, weakness, fainting) led to a medical consultation
    b) The condition was detected incidentally during examinations (e.g., ECG, pulse check, medical visit for other reason)
    c) I do not remember
11. **How would you rate the severity of your atrial fibrillation or atrial flutter symptoms (EHRA scale)**
    a) No symptoms
    b) Mild symptoms – do not affect daily activities
    c) Moderate symptoms – bothersome but tolerable
    d) Severe symptoms – limit daily activities
    e) Symptoms disabling normal functioning
12. **How often do episodes of atrial fibrillation or atrial flutter occur?**
    a) Less than once a month
    b) Several times a month
    c) Several times a week
    d) Daily
    e) I do not experience symptoms of arrhythmia
13. **How do you manage when an arrhythmia episode occurs?**
    a) I do not experience arrhythmia
    b) I immediately call emergency medical services or go directly to a hospital or outpatient clinic
    c) I take additional medication, and if it does not help, I seek medical assistance
14. **Have you ever had a stroke?**
    a) Yes
       1. Hemorrhagic stroke - please specify the year of occurrence or how long ago it occurred (in years) ........................................................
       2. Ischemic stroke – please specify the year of occurrence or how long ago it occurred (in years) ......................................................................
    b) No
15. **To what extent do you adhere to the following medical recommendations? (please mark an “X” in the appropriate box):**

|  | Not at all | To a minimal extent | To a slight extent | To a moderate extent | To a great extent | Not applicable |
| --- | --- | --- | --- | --- | --- | --- |
| Regular medication intake |  |  |  |  |  |  |
| Maintaining a healthy body weight |  |  |  |  |  |  |
| Avoiding excessive alcohol consumption and/or smoking |  |  |  |  |  |  |
| Engaging in physical activity |  |  |  |  |  |  |
| Monitoring and managing:   - Blood pressure - Blood glucose level - Obstructive sleep apnea - Cholesterol level |  |  |  |  |  |  |

16. **Which of the following statements best describes you?**
    a) I regularly take my prescribed medications
    b) I most often forget my evening medications
    c) I frequently forget to take my medications
    d) I adjust my medication doses myself depending on blood pressure and heart rate measurements
17. **How much of a problem is taking anticoagulant medication for you?**
    a) I have no problem with it
    b) I am worried that I might bleed excessively by accident
    c) I feel calm because I know it helps prevent ischemic stroke
    d) I am aware of the risks and benefits, and therefore I take these medications regularly
18. **Have you ever been diagnosed with mental health problems?**
    a) Yes
    b) No
19. **Have you received psychiatric treatment within the last two years?**
    a) Yes
       1. If yes, please specify the reason…
    b) No
20. **Have you visited a psychologist in the past two years?**
    a) Yes
    b) No
