# Supplementary material for: An analysis of the relationship of selected personality traits on perceived stress in patients undergoing ablation for atrial fibrillation and atrial flutter
Source: Front Psychiatry. 2026 Aug 10;17:1681576. doi: 10.3389/fpsyt.2026.1681576 (PMC13501273; doi:10.3389/fpsyt.2026.1681576)
Supplement: Supplementary file 3 [file Table3.docx]

STROBE Statement—Checklist of items that should be included in reports of ***cross-sectional studies***

Manuscript title: An analysis of the relationship of selected personality traits on perceived stress in patients undergoing ablation for atrial fibrillation and atrial flutter

|  | Item No | Recommendation |
| --- | --- | --- |
| **Title and abstract** | 1 | The abstract includes information on the study design, study objectives and background, methods, results, and conclusions. |
| Introduction | | |
| Background/rationale | 2 | The scientific background and rationale for the study are described in the Introduction section. |
| Objectives | 3 | The specific objectives of the study are provided in the Introduction section (lines 123-126). No prespecified hypotheses were formulated. |
| Methods | | |
| Study design | 4 | The key elements of the study design are described in the Abstract and the Materials and Methods section. |
| Setting | 5 | The setting, locations and relevant dates are described in the Materials and Methods section (lines 130-132; 134-137). |
| Participants | 6 | The eligibility criteria and methods of participants selection are described in the Materials and Methods section (lines 137-141). |
| Variables | 7 | The outcomes and exposures are defined in the Materials and Methods section (lines 141-145). The potential predictors, confounders, and effect modifiers were assessed by the custom-made questionnaire provided in the Supplementary Materials. This information is stated in the Materials and Methods section (lines 145-155) |
| Data sources/ measurement | 8* | Details regarding the sources of data and methods of assessment for each variable are provided in Sections 2.1, 2.2. and 2.3. |
| Bias | 9 | Efforts to address potential sources of bias are described in the Materials and Methods section (lines 155-159). |
| Study size | 10 | The explanation of the study size is described in Materials and Methods section (lines 132-134). |
| Quantitative variables | 11 | The explanation of how the quantitative variables were handled is presented in Section 2.4. |
| Statistical methods | 12 | The explanation of all statistical methods is presented in Sections 2.4 and 3.1. References to the missing data and sensitivity analyses are not applicable in this study. |
| Results | | |
| Participants | 13* | The study is single staged. The number of participants is given in Section 3.1 (line 190). |
| Descriptive data | 14* | The characteristics of study group as well as information on exposures and potential confounders are given in Section 3.1 and Table 1. References to the missing data are not applicable in this study. |
| Outcome data | 15* | The Results section presents summary measures for all study variables, including descriptive statistics, correlation coefficients, comparisons and regression coefficients with corresponding significance levels. |
| Main results | 16 | All relevant information regarding unadjusted and adjusted estimates, measures of precision, confounders included in the analyses, are presented in the Results Section and the corresponding tables. Subpoint b and c are not applicable in this study. |
| Other analyses | 17 | The analyses of any subgroups and interactions are provided in Sections 3.2, 3.3 and 3.4 |
| Discussion | | |
| Key results | 18 | The key results are presented Discussion section (lines 233-293) with overall interpretation |
| Limitations | 19 | The limitations of the study are discussed in Discussion section (lines 306-327) |
| Interpretation | 20 | The key results are presented Discussion section (lines 233-293) with overall interpretation |
| Generalisability | 21 | The generalizability of the study results is discussed in the Conclusion section |
| Other information | | |
| Funding | 22 | Not applicable |

*Give information separately for exposed and unexposed groups.

**Note:** An Explanation and Elaboration article discusses each checklist item and gives methodological background and published examples of transparent reporting. The STROBE checklist is best used in conjunction with this article (freely available on the Web sites of PLoS Medicine at http://www.plosmedicine.org/, Annals of Internal Medicine at http://www.annals.org/, and Epidemiology at http://www.epidem.com/). Information on the STROBE Initiative is available at [www.strobe-statement.org](https://www.strobe-statement.org).
